# Prognostic Factors in Patients with Multiple Recurrences of Well-Differentiated Thyroid Carcinoma

**DOI:** 10.1155/2009/650340

**Published:** 2009-10-15

**Authors:** Theresa Holler, Jenna Theriault, Richard J. Payne, Jonathan Clark, Spiro Eski, Jeremy L. Freeman

**Affiliations:** Department of Otolaryngology—Head & Neck Surgery, University of Toronto, Toronto, ON, Canada

## Abstract

*Introduction.* Patients with multiple recurrences of well-differentiated thyroid carcinoma (WDTC) have markedly reduced overall survival when compared with those who have ≤1 recurrence of their disease. The purpose of this investigation is to identify prognostic factors for mortality in this subgroup. *Methods.* Patients with multiple recurrences of WDTC were retrospectively identified from the thyroid cancer database at Mount Sinai Hospital, Toronto (1963–2000). Data on patient, tumor, and recurrence characteristics were collected, and each patient was given a MACIS score. *Results.* A total of 31 patients were identified (11 male, 20 female; 16–83 years). Using univariate analysis, age >45, stage III/IV disease, distant metastasis, vascular invasion, MACIS score >6, and time to recurrence of <12 months were found to be significant predictors for mortality in this subgroup. *Conclusions.* Patients with multiple recurrences of WDTC follow a distinct clinical course, marked with multiple treatment failures and a substantial risk of mortality.

## 1. Introduction

Although well-differentiated thyroid carcinoma (WDTC) is the most commonly diagnosed thyroid malignancy, it accounts for only 2% of all cancers in the body and is responsible for less than 0.5% of cancer-related deaths [1]. Combination therapy with thyroidectomy and adjuvant I^131^ is the treatment of choice at most institutions. The majority of patients have an excellent prognosis, with disease-specific survival rates at 10 years greater than 90% [1]. However, 8%–23% of patients will fail initial therapy and go on to develop a recurrence of their disease [1–4]. Mortality rates among patients with disease recurrence have been reported to be as high as 38%–69% [4–6]. In a previous study, Palme et al. [7] showed that WDTC patients who had either no recurrence of their disease or only one recurrence after initial therapy had no difference in disease-specific or overall survival (100% versus 94%, 89% versus 83%, resp.). In addition, patients with multiple treatment failures had significantly reduced survivals (60% and 58%, resp., *P* < .001). 

There has been a large body of research compiled over the past few decades examining various prognostic factors for both recurrence and mortality in patients with WDTC. Factors such as age >45, male sex, large tumor size, histological type, advanced stage of disease, extrathyroidal extension, lymphatic invasion, and presence of distant metastasis have all been cited as indicators of poor outcome [1–4, 6, 8–20]. Several groups have attempted to classify patients into low-, intermediate-, and high- risk groups based on the presence of these factors [10, 17]. Prognostication is therefore used to identify patients at high risk who require close follow-up and prompt therapy for any evidence of disease recurrence. To our knowledge, there are no reports in literature delineating prognostic factors to predict disease outcome in patients who have suffered with multiple treatment failures of WDTC. Thus, the purpose of this investigation is to examine patient, tumor, treatment, and recurrence factors that may predict for mortality among patients with multiple recurrences of WDTC.

## 2. Materials and Methods

Thirty-one patients with multiple recurrences of WDTC were retrospectively identified from the thyroid cancer database at Mount Sinai Hospital, Toronto (1963–2000). Recurrence was defined as any evidence of disease requiring further treatment after initial curative therapy. Patient (age, sex), tumor (histology, size, stage, solitary/multifocal, extrathyroidal spread, vascular invasion, lymphatic invasion), and treatment (extent of initial surgery, adjuvant I^131^, and external beam radiation) characteristics were collected. Information about the site of each recurrence (local, regional, distant, unspecified), mode of detection (clinical, imaging, thryoglobulin estimation), and treatment (surgery, I^131^ therapy) were also recorded. Extent of disease at presentation was staged according to the American Joint Committee on Cancer (AJCC) staging system for WDTC [21]. In addition, each patient was scored according to the Metastasis, Age, Completeness of Resection, Invasion, and Size (MACIS) prognostic index [22]. Extent of initial surgery was recorded as either a subtotal or total thyroidectomy with or without an accompanying neck dissection. A recurrence was classified as unspecified if thyroglobulin levels were elevated in the presence of a negative clinical exam and failure of localization with available imaging modalities (i.e., ultrasonography, I^131^ scanning, CT, and MRI). Final outcome was recorded as alive, no evidence of disease (ANED), alive with disease (AWD), dead, no disease (DND), and dead of disease (DOD). Follow-up was counted from completion of initial therapy to the last known clinical encounter or date of death. 

Statistical analysis of survival data was performed using the Kaplan-Meier method, and curves were compared using the log-rank test. *P* < .05 was considered statistically significant. Univariate analysis was performed in order to identify prognostic factors significant for the development of a poor outcome (i.e., death) in patients with multiple recurrences of WDTC. Multivariate analysis using the Cox proportional hazards model was not possible due to the limited number of events in this study. All statistics were carried out using SPSS software (SPSS Inc, Chicago, Ill).

## 3. Results

Thirty-one patients with multiple recurrences of WDTC were identified from treatment records at the Department of Otolaryngology — Head & Neck Surgery, Mount Sinai Hospital (Toronto), with a median follow-up of 12.6 years (range 9 months–35.5 years). There were 20 (64.5%) female patients and 11 (35.5%) male patients (median age 43, range 16–83 years; Table 1). The final histopathologic diagnosis was papillary carcinoma in 19 (61.3%), tall cell variant in 5 (16.1%), follicular carcinoma in 4 (12.9%), and mixed in 3 (9.7%) cases. The median size of the dominant nodule was 3.3 cm (range 0.5–5.5 cm). Seven of the charts did not contain a report of tumor size and thus could not be scored with the MACIS prognostic index. These patients were excluded from further analysis. Of the 31 patients identified, 6 (19.4%) had evidence of distant metastasis at diagnosis. Other tumor characteristics present at first surgery included multifocal disease in 21 (67.7%) patients, extrathyroidal spread in 18 (58.1%) patients, lymphatic invasion in 18 (58.1%) patients, and vascular invasion in 3 (9.7%) patients. According to the AJCC staging system, Stage I disease was present in 4 patients (12.9%), Stage II in 8 patients (25.8%), Stage III in 11 patients (35.5%), and Stage IV in 8 patients (25.8%). The MACIS prognostic index was applied to 24 cases in the series with complete pathology records (median score 6.03, range 3.25–11.82). 

Extent of initial surgery was dependent on both disease severity and the prevailing treatment philosophy at the time of diagnosis. A total thyroidectomy was performed in 19 (61.3%) patients, whereas a subtotal thyroidectomy was performed in 12 (38.7%) cases. A neck dissection accompanied thyroidectomy in 15 patients with evidence of nodal metastasis at initial surgery. Almost all patients in this series were treated with adjuvant I^131^ (87.1%). Ten patients had residual disease severe enough after initial operation to warrant external beam radiation therapy (ERT). 

All patients in this series experienced multiple treatment failures. The average time to first recurrence was 25.4 months (range 0.2–185.4 months). Recurrences were classified as local (19.4%), regional (48.4%), distant (19.4%), and unspecified (12.9%). Neither the mode of detection (i.e., clinical, imaging, or elevated thyroglobulin) nor the method of treatment (surgery, I^131^, or ERT) for recurrent disease was found to be a significant predictor of survival in this study population.

**Figure 1:**
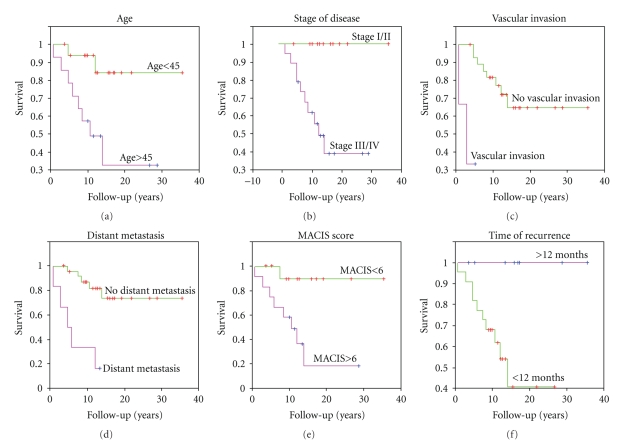
Prognostic factors significantly associated with mortality in patients with multiple recurrences of well-differentiated thyroid carcinoma. (a). age >45, (b). stage III/IV disease, (c). angioinvasion, (d). distant metastasis, (e). MACIS score >6, (F). time to recurrence <12 months.

Thirty-two percent of patients with multiple recurrences of WDTC died of their disease (DOD). Other outcomes included alive, no evidence of disease (ANED) in 29% and alive with disease (AWD) in 38.7%. No patients in this series died of causes unrelated to their thyroid carcinoma. Actuarially predicted disease-specific survival among patients with multiple treatment failures at 20 years was 60%, a significant reduction from that of patients with either no recurrences or only one recurrence of their disease [7]. Univariate analysis revealed that age >45, stage III/IV disease, distant metastasis, vascular invasion, MACIS score >6, and a time to recurrence of <12 months are all predictive factors for mortality in this group (*P* < .01, < .01, < .001, < .001, < .01, < .03, resp.; Figure 1). In addition, gender, histological type, initial surgery (total thyroidectomy vs. subtotal thyroidectomy), initial I^131 ^therapy, multifocal disease, tumor size, lymphatic invasion, and neck dissection were shown to have no predictive utility in this subgroup of patients with WDTC (Table 2).

## 4. Discussion

Despite optimal treatment, patients with WDTC often experience disease recurrence, with rates reported in literature ranging from 8% to 23% [1–4]. Palme and associates [7], reported no significant difference in disease specific and overall survival among WDTC patients cured after initial therapy, and those with a single recurrence. However, patients with multiple recurrences are at a significantly increased risk of death, with mortality rates ranging between 12% and 69% [2, 4–6]. It appears that patients who develop multiple recurrences of WDTC follow a distinct course, marked by multiple treatment failures and a significant risk of mortality. It was the intention of the present study to delineate patient, tumor, treatment, and recurrence factors that may be used by physicians to predict for mortality in these patients. 

A large body of research exists exploring various prognostic factors for recurrence and mortality among patients with WDTC. Characteristics such as age, sex, tumor size, stage, extrathyroidal spread (ETS), nodal metastases, distant metastases, and extent of initial surgery have all been cited as indicators of poor outcome [1–4, 6, 8–20]. In this investigation, we found that among patients with multiple treatment failures, age >45, stage III/IV disease, distant metastasis, vascular invasion, MACIS score >6, and a time to recurrence of <12 months predicted mortality in this group. 

Several studies have cited advanced age as being one of the most significant predictors for recurrence and mortality among patients with WDTC. Shah et al. [8] reported that patients above the age of 50 have a 270% greater risk of death from differentiated thyroid cancer than their younger cohorts. In addition, several authors have cited that the greatest change in prognosis occurs at the age of 45, with older patients having significantly reduced total survival [6, 8–13]. This is in agreement with the results of the current study; patients with multiple recurrences of WDTC who are older than 45 years of age are at an increased risk of death from their disease (*P* < .01). 

Extent of disease at initial diagnosis strongly influences prognosis in patients with WDTC. Large tumor size, especially >4 cm, has been shown to adversely affect mortality in multiple trials [9–11, 14]. Although greater tumor size (i.e., >4 cm) was found to be a predictor of multiple recurrences in patients with WDTC [7], it did not appear to predict for mortality in this population. 

The AJCC staging system, which incorporates the TNM (tumor, lymph nodes, metastases) classification, is the current standard in staging thyroid malignancies. Stage III/IV disease (III = ETS or nodal metastases, IV = distant metastases) appears to portend an increased risk of mortality among patients with multiple recurrences of WDTC. This is in agreement with other authors, who have found that advanced stage disease not only increases the risk of recurrence but aslo significantly reduces disease-specific survival [7–9, 12, 13, 15]. In the present study, there appears to be a trend toward significance for the adverse effect of extrathyroidal extension (T3) on mortality (*P* = .07). In addition, we found that neither lymphatic invasion nor initial neck dissection showed a statistical significance for mortality among patients with WDTC. However, the presence of vascular invasion did appear to portend a poor prognosis on survival in this cohort (*P* = .002). Lastly, distant metastases were found to be a highly significant predictor of mortality among patients with multiple recurrences of WDTC (*P* = .0002). 

Patients who have their first treatment failure <12 months after initial therapy appear to have significantly shorter survival than those who recur after one year. The median time to first recurrence in the present study was 7.3 months, with one patient not showing evidence of any treatment failure until more than 15 years after initial surgery. Given the extensive length of time which may pass between initial treatment and recurrence, life-long follow up is necessary. 

Several authors have shown that both the method of detection and the treatment modality used for a first recurrence can predict future treatment failures [7, 20]. That is, patients who have clinically detectable disease recur at a greater rate than those whose first recurrence is detected by thyroglobulin measurement or imaging modalities, stressing the need for early detection before tumor burden becomes significant. In the present investigation, neither the method of detection (i.e., clinical, imaging, I^131^, thyroglobulin) nor the treatment modality (surgery, I^131^, external beam radiotherapy) influenced the mortality rates among patients with multiple treatment failures of WDTC. 

Because both multiple recurrences and mortality from WDTC are a rare event, it was necessary in the present study to collect data over several decades. In 37 years of clinical experience treating WDTC at our institution, only 31 patients were identified as having multiple recurrences of their disease. Of these, only 10 patients died from their disease. In addition, it is well known that patients with WDTC can be free from disease for many years before developing a first recurrence, a phenomenon that necessitates life-long follow-up in these patients. The long duration of follow-up in the present study allows for the identification of disease recurrences as well as an assessment of long-term clinical outcomes. 

Despite changing treatment paradigms over the 4 decades analyzed in this study, all 10 patients who died of their disease had both surgical and adjunctive management of their initial disease that is comparable to current practices at our institution. All patients with advanced stage WDTC underwent either a total thyroidectomy (*n* = 8) or a completion thyroidectomy at the time of initial diagnosis (*n* = 2). Those with unresectable gross residual disease were treated with external beam radiation therapy. Patients who were considered to have microscopic residual disease or who had advanced stage disease were given postoperative I^131^ ablation. In 2002, Hay et al. reported 6 decades of experience treating papillary thyroid carcinoma. They found that despite evolving treatment paradigms, there was no difference in overall survival among patients treated with subtotal versus total thyroidectomy and no survival benefit to postoperative I^131^ ablation in low-risk patients (MACIS score <6) [23]. Given this, it is unlikely that changing treatment paradigms has significantly influenced the validity of the current results.

## 5. Conclusions

To our knowledge, this is the first paper to identify prognostic factors among patients with well-differentiated thyroid carcinoma who suffer multiple recurrences of their disease; patients with multiple recurrences of WDTC follow a poor clinical course, with multiple treatment failures and decreased survival. Among this subgroup, those aged 45 years or more with aggressive primary tumors (ETS and vascular invasion) and advanced stage disease (Stage III/IV, MACIS>6) have a significantly higher risk of mortality. In addition, time to first recurrence within 12 months of initial therapy conveys a worse prognosis. Interestingly, mortality rates in this study were not influenced by the method of detection nor the type of therapy chosen for first recurrences. One potential explanation is that these patients have a more biologically aggressive variant of WDTC which does not readily respond to treatment of the primary tumor or the initial recurrence. Although the failure to cure these cases after several attempts may cause frustration in both the treating physician and the patient, close follow-up and aggressive treatment of further recurrences is still warranted, as approximately 30% of these will go on to be free of disease after subsequent therapies. Further research is needed into the biological and molecular markers of tumor severity in order to provide an understanding of why some patients with WDTC have an excellent prognosis with complete cure, while others are plagued by multiple treatment failures and eventual death.

## Figures and Tables

**Table 1:** Demographic data for patient with multiple recurrences of well-differentiated thyroid carcinoma.

N	31
Age, median (range), years	43 (16–83)
Sex, M:F	11:20
Histologic type	
* *Papillary	19
* *Follicular	4
* *Mixed	3
* *Tall cell	5
Tumour size, median (range), cm	3.3 (0.5–5.5)
Multifocal disease	21
Extrathyroidal extension	18
Lymphatic invasion	18
Vascular invasion	3
Distant metastasis	6
AJCC stage	
* *I	4
* *II	8
* *III	11
* *IV	8
MACIS, median (range)	6.03 (3.25–11.02)
Extent of initial surgery	
* *Subtotal thyroidectomy	12
* *Total thyroidectomy	19
* *Neck dissection^†^	15
Lodine 131 therapy	27
External beam radiation therapy	10
Site of recurrence	
* *Local	6
* *Regional	15
* *Distant	6
* *Unspecified	4
Outcome	
* *Alive, no evidence of disease	9
* *Alive with disease	12
* *Dead of disease	10
* *Dead, no evidence of disease	0
Follow–up, median (range), years	12.6 (9 months 35.5 years)

AJCCAmerican JOINT Committee on Cancer. Data is for the number of patients in each category. ^†^central +/- lateral neck dissection. Unspecified recurrence-elevated thyroglobulin levels in the presence of a negative clinical exam and failure of localization with imaging modalities (i.e., ultrasonography, I131 scanning, CT and MRI).

**Table 2:** Patient, tumour, treatment, and recurrence data for patients with multiple recurrences of well-differentiated thyroid carcinoma.

Age > 45	**P = .0093
Gender (M Versus F)	*P* = .7876
Extent of initial surgery (ST versus TT)	*P* = .0964
Neck dissection	*P* = .1978
Iodine 131 therapy	*P* = .1749
External beam radiation therapy	*P = .0167
Histologic type—overall	*P* = .1362
* *Tall cell versus others	^†^ *P* = .0748
Stage I/II versus IV	**P = .0052
Size > 4 cm	^†^ *P* = .0621
Multifocal disease	^†^ *P* = .0795
Lymphatic invasion	*P* = .9361
Vascular invasion	***P = .0002
Extrathyriodal extention	^†^ *P* = .0772
Distant metastasis	***P = .0002
MACIS > 6	**P = .0061
Time to recurrence < 12 months	*P = .0270
Site of recurrence	^†^ *P* = .0560
Mode of detection	
* *Clinical	*P* = .6328
* *Imaging	*P* = .5752
* *Thyroglobulin	*P* = .7497
Treatment of recurrence	
* *Surgery	*P* = .4128
* *Iodine 131 therapy	*P* = .6281
* *External beam radiation therapy	*P* = .8889

ST: subtotal thyroidectomy;*   *TT: total thyroidectomy *P < .05;*  ***P < .01;*  ****P < .001 ^†^ trend toward significance.
